# Biocomposites Based on Mould Biomass and Waste Fibres for the Production of Agrotextiles: Technology Development, Material Characterization, and Agricultural Application

**DOI:** 10.3390/ma17246084

**Published:** 2024-12-12

**Authors:** Beata Gutarowska, Dominika Gibka, Konrad Olejnik, Piotr Pospiech, Tomasz Boruta, Tomasz Kapela, Krzysztof Makowski

**Affiliations:** 1 Department of Environmental Biotechnology, Faculty of Biotechnology and Food Sciences, Lodz University of Technology, Wólczańska 171/173, 90-530 Łódź, Poland; 2 Biotechnika Poland Sp. z o.o., Tymienieckiego 25, 90-350 Łódź, Poland; d.gibka@biotechnika.net (D.G.); t.kapela@biotechnika.net (T.K.); k.makowski@biotechnika.net (K.M.); 3 Centre of Papermaking and Printing, Lodz University of Technology, Wólczańska 221, 93-005 Łódź, Poland; konrad.olejnik@p.lodz.pl (K.O.); piotr.pospiech@p.lodz.pl (P.P.); 4 Department of Bioprocess Engineering, Faculty of Process and Environmental Engineering, Lodz University of Technology, Wólczańska 213, 93-005 Łódź, Poland; tomasz.boruta@p.lodz.pl

**Keywords:** mould biomass, plant cellulosic fibres, waste fibres, biocomposites, agrotextiles

## Abstract

This study explores the potential use of mould biomass and waste fibres for the production of agrotextiles. First, 20 mould strains were screened for efficient mycelium growth, with optimized conditions of temperature, sources of carbon and nitrogen in the medium, and type of culture (submerged or surface). A method was developed for creating a biocomposite based on the mould mycelium, reinforced with commercial bleached softwood kraft (BSK) pulp and fibre additives (cotton, hemp). The best properties, including mechanical, water permeability, and air permeability, were shown by the biocomposites containing 10–20% *Cladosporium cladosporioides* mycelium grown in surface or submerged cultures, milled with BSK pulp, cotton, and hemp (10–20%). The mould mycelium was refined with cellulosic fibrous material, formed, pressed, and dried, resulting in a biomaterial with good mechanical parameters, low water permeability, and high air permeability. The biocomposite was fully biodegradable in soil after 10 days in field conditions. The use of the biocomposite as a crop cover shortened the germination time and increased the percentage of germinated onion, but had no effect on parsley seeds. This study shows the potential of using mould mycelium for the production of biomaterial with good properties for applications in horticulture.

## 1. Introduction

The growing interest in sustainability has driven the search for raw materials that align with the principles of a circular economy. This approach emphasizes minimizing waste, maximizing resource efficiency, and promoting the reuse and recycling of materials to create a more sustainable production and consumption system. In this context, mycelium-bound biocomposites reinforced with waste-based additives offer a promising material with a wide range of possible applications in modern material technologies [1]. The Basidiomycota phylum, including the genera *Ganoderma*, *Pleurotus*, *Trametes*, *Schizophyllum*, *Lentinula*, and *Fomes*, plays a crucial role in fungal-based material technologies. Notable species within these genera include *Ganoderma lucidum*, *Ganoderma oregonense*, *Pleurotus ostreatus*, *Pleurotus djamor*, *Trametes versicolor*, *Schizophyllum commune*, *Lentinula edodes*, and *Fomes fomentarius*. These white and brown rot fungi are capable of colonising and degrading various substrates containing cellulose, hemicelluloses, and lignin [2,3,4,5]. An important consideration in the design of fungal-based biocomposites is the choice of substrate used for mycelial growth. Various substrates can be used, including cereal straws, wood sawdust, or other fibres, such as flax and cotton. During growth, mycelium filaments enclose and degrade the material, binding the digested substrate [6]. Fungal mycelium thus provides a natural “glue”, forming a solid material [7]. Depending on the fungus species, substrate, growth conditions, additives, and processing methods, the resulting biocomposite can exhibit diverse properties [8,9]. Fungal-based materials have potential applications in a wide range of fields, including biomedicine and cosmetics (e.g., antimicrobial wound healing patches based on mycelium with curcumin, tissue engineering adhesion platforms), packaging (e.g., MycoFoam; EcoCradle, Ecovative Design), the textile and leather industry (clothing, footwear, bags), the pulp and paper industry, the furniture and construction industries (mycelium bricks, structural frameworks, thermal and acoustic insulation panels, e.g., MycoFlex™; MycoComposite™; Mycotecture; Mycotecture Alpha; Mycelium + Timber series; MycoTree; Mycelium Chair), and electronics [4,10,11,12,13,14,15,16,17].

Due to the many parameters and variables in the production of mycelium-associated biocomposites, their full potential has yet to be realised [6]. While there has been extensive research on higher fungal-based biocomposites with applications in the textile and paper industries, the production of mould mycelium-based biocomposites has not been reported widely in the literature. To date, only a few mould species, such as *Acremonium* sp. and *Trichoderma asperellum*, have been used in consortia with white rot fungi for the production of biocomposites [17]. Examples described in the literature include a biocomposite based on a consortium of the higher fungi *Pleurotus ostreatus*, *Oudemansiella radicata*, and the mould *Acremonium* sp. on a mixed substrate of cotton stalks, wheat bran, and natural reinforcing particles [17]. Jones et al. [18] produced polymer extracts and nanopapers from different species of higher fungi and the mould *Mucor genevensis* grown on sugarcane molasses. The nanopapers showed high tensile strength properties and hydrophobicity, making them suitable for a wide range of applications, including coatings, membranes, packaging, and paper. The biocomposites obtained in the study have potential applications as a lightweight infill material for geotechnical engineering [19].

Moulds are already widely used in the food industry for cheese production, in the dairy industry, and in the fermentation of soy-based products such as miso or soy sauce. Moulds are also used in the production of enzymes (amylases, pectinases, cellulases), acids, and biocontrol agents, as well as in the pharmaceutical industry for the production of antibiotics (e.g., penicillin) [20]. Some species of higher fungi, including *Ganoderma lucidum* with various substrates, are used in the textile industry for the biofabrication of leather-like materials [21]. The mycelium is thermally or chemically inactivated, pressed, and extruded into products resembling real leather. Textiles made with higher fungal mycelium are waterproof, fireproof, and non-toxic. To obtain highly flexible fibre mycelial mats, they can be pre-treated with lubricants or plasticisers such as glycerol, polyethylene glycol, or sorbitol [22]. In the paper industry, an interesting application of mycelium biocomposites is the production of paper from pulp derived from mycelium grown using lignocellulosic substrates or wood by-products [23]. Mycelial paper is produced in a similar way to conventional papermaking by soaking the mycelium in water, mixing it to obtain a pulp, and then dehydrating and drying the biocomposite. Mycelium-based biocomposites contain the structural polysaccharide chitin, and paper made from mycelium biomass can compete with wood-based paper [18]. Due to the widespread use of moulds in various industries, significant amounts of waste mycelium are generated, necessitating effective management solutions. Waste mycelium has a wide range of possible applications, from bioremediation and composting to the production of functional materials such as acoustic and thermal insulation and biodegradable packaging [24,25,26].

Moulds and higher fungi can grow on any substrate in the form of mycelium. This growth is faster in the case of mould (a few days) than in the case of higher fungi (weeks), however, moulds produce less mycelium biomass. Mycelium primarily consists of natural polymers such as chitin, cellulose, proteins, etc., making it a natural polymeric composite fibrous material. The cell wall composition of mycelium varies among the different species of fungi. Typically, the cell wall structure of fungi comprises mainly mannoproteins, β-glucans, chitin (C_22_H_54_N_21_)n, and, in some cases, cellulose [27]. The structure and chemical composition of mycelium, which is rich in carbon, nitrogen, oxygen, and other microelements such as phosphorus and sulphur, give it unique mechanical and biological properties that can be easily degraded in the environment [28].

In the context of a circular economy, the use of mould mycelium as a raw material supports the idea of closing the product life cycle [29]. In particular, mould mycelium offers a substitute for plastics and synthetic materials, which are becoming increasingly unacceptable environmentally and have a negative impact on ecosystems [30]. One of the key challenges facing contemporary agriculture is to cease using synthetic agrotextiles. Synthetic agrotextiles are used in agriculture for crop protection, soil stabilization, weed control, water management, and greenhouse applications. However, they generate significant soil pollution and microplastic emissions, as well as having detrimental effects on plant cultivation [31]. Increasingly, biodegradable nonwovens made from biopolymers and products incorporating plant additives and animal bioproducts, such as wool, are seen as alternatives to synthetic agrotextiles, reducing negative impacts on ecosystems while offering functional properties such as biodegradability and favourable mechanical characteristics [32]. However, there are no studies in the literature on the production of agrotextiles based on mould mycelium.

The aim of this research was to develop a method for producing a biocomposite based on mould mycelium and plant fibrous materials, in the form of flat sheets for agricultural applications. Specific objectives included the following: (1) the screening of fast-growing moulds capable of producing abundant mycelium; (2) determining optimal growth conditions for the selected strain; (3) designing the composition and production method of the biocomposite sheet based on the inactivated mycelium and fibrous additives; (4) evaluating the properties of the obtained biomaterial, including strength parameters, hydrophobicity, and the biodegradation rate under controlled and field conditions; and (5) evaluating the use of the biocomposite agrotextile for seed germination.

## 2. Materials and Methods

### 2.1. Screening of Moulds

The following mould strains (n = 20) from the collection of the Department of Environmental Biotechnology, Faculty of Biotechnology and Food Sciences, Lodz University of Technology, were used for the screening study: *Alternaria* sp. (2 strains), *Aspergillus flavus*, *Aspergillus fumigatus*, *Aspergillus niger*, *Aureobasidium* sp. (2 strains), *Cladosporium* sp. (2 strains), *Cheatomium globosum*, *Cheatomium* sp., *Fusarium* sp., *Fusarium oxysporum*, *Mucor* sp., *Penicillium* sp. (2 strains), *Rhizopus* sp., *Scopulariopsis* sp., *Trichoderma viride*, and *Ulocladium* sp.). The selection criteria for the strains were potential industrial applications, high biomass yield, growth rate in submerged cultures, and good growth in surface cultures. Submerged cultures were grown on MEB (malt extract broth; Merck, Darmstadt, Germany) medium, followed by inoculation with 4% (*v*/*v*) mould suspension (10^6^ CFU/mL colony-forming units per ml) in physiological saline (0.85% solution). Cultivation was carried out for 120 h at temp. 22 ± 2 °C with continuous shaking at 160 rpm (ChemLand, SK-O330-PRO, Stargard, Poland). The growth of the mould was determined using the gravimetric method. Flasks were weighed using a balance (AS.310.R2Plus, Radwag, Radom, Poland) immediately after inoculating the media with mould suspensions and then after each 24 h of cultivation. This approach allowed the dynamics of mycelial biomass growth to be determined during cultivation. After 120 h of cultivation, the biomass was deactivated (at temp. 121 °C, 15 min), filtered, and the yield of dry biomass (dried at 105 °C) was determined. Surface cultivation was conducted on MEA (Merck, Germany) medium for 120 h at temp. 22 ± 2 °C. Growth was estimated on the basis of morphological observation of colonies. After screening, strain No. 8 (*Cladosporium* sp.) was selected and identified as the species *Cladosporium cladosporioides.*

The taxonomic affiliation of the mould was determined based on the sequence of the ITS rDNA region. Isolation of genomic DNA was performed using a Soil DNA Purification Kit (EURx, Gdańsk, Poland). The following primers were used: ITS1–TTC GTA GGT GAA CCT GCG G; ITS4–TCC TCC GCT TAT TGA TAT GC. The PCR reaction was carried out in a volume of 50 µL. The reaction mixture consisted of 5 µL (~50 ng) of genomic DNA isolated from the sample, 25 µL of 2× Colour Opti Taq PCR master mix (A EURx, Poland), 0.5 L of 10 mM ITS1 primer, 0.5 L of 10 mM ITS4 primer, and 19 µL of sterile water. The reaction was carried out under the following conditions in five stages: I. (95 °C, 3 min), II. 34 cycles (95 °C, 30 s), III. (58 °C, 30 s), IV. (72 °C, 1 min), V. (72 °C, 7 min). DNA fragments obtained from the amplification reaction were purified using a DNA Mini Kit (Syngen, Wrocław, Poland) and sequenced at Genomed (Warszawa, Poland). The obtained nucleotide sequences were analysed and compared with sequences published in the National Centre for Biotechnology Information (NCBI) database using BLASTN 2.13.0+ software.

### 2.2. Evaluation of Culture Conditions for Moulds

The following parameters were used to evaluate the mould culture conditions: temperature, shaking speed, source of carbon and nitrogen in the medium, and type of culture (submerged in a bioreactor or surface cultures). To evaluate biomass growth at different temperatures (20, 25, and 28 °C), cultivation was carried out in Erlenmeyer flasks in MEB medium (pH: 4.8 ± 0.2), inoculated with 4% (*v*/*v*) *C. cladosporioides* inoculum (10^6^ CFU/mL) for 120 h at 190 rpm (Thermo Scientific, Heraeus, Boston, MA, USA). Cultivation was also conducted at three different shaking speeds: 130, 160, and 190 rpm (ChemLand, SK-O330-PRO, Poland) at 28 °C for 120 h. To evaluate the carbon source, the following components were used: sucrose (Chempur, Piekary Śląskie, Poland), glucose-fructose syrup (Fractal Colour, Bydgoszcz, Poland), maltodextrins (Liniaplus, Topolin, Poland), and beet molasses (Harison, Warszawa, Poland). The medium was prepared with three concentrations of each carbon source: 40 g/L, 60 g/L, and 100 g/L. As a nitrogen source for mould growth, 5 g/L of casein peptone tryptone (BTL, Warsaw, Poland) was used. To evaluate the nitrogen source, soy peptone (Pol-Aura, Olsztyn, Poland) and yeast extract (JHJ, Gizałki, Poland) were used in the medium in concentrations of 10 g/L, 20 g/L, and 50 g/L. Beet molasses was used as a carbon source at a concentration of 60 g/L. Cultivation was carried out at 28 °C for 120 h at 190 rpm.

Fungal growth in the form of pellets was observed whenever shaking or stirring of the cultivation broth was applied. Pellets are spherical compact aggregates of mycelium often observed in submerged cultures of fungi [33,34]. To evaluate biomass production in submerged (bioreactor-based) and surface cultures (on Petri dishes), three stirred-tank bioreactors (Biostat B, Sartorius, Göttingen, Germany) were used, with a total volume of 6.6 L. The cultivation processes using bioreactors were conducted with a working volume of 5 L of the liquid medium. The liquid medium (60 g/L beet molasses, 10 g/L soy peptone, pH: 4.8) was sterilized in an autoclave at 121 °C. The inoculum was prepared by washing the spores of *C. cladosporioides* from MEA slants into sterile liquid medium with the use of a 1 mL plastic pipette. The bioreactor cultures were initiated at a 5% (*v*/*v*) inoculum ratio (10^6^ CFU/mL). The temperature (28 °C) and air flow rate (2.5 L_air_/min) were kept constant throughout the cultivation process. The three bioreactors operated at constant stirring speed values of 200, 275, or 350 rpm, respectively. The bioreactor cultures were propagated for 120 h. Antifoam SE-15 solution (Merck, Germany) was used to control foaming during the process. Surface cultivation was conducted on large Petri dishes with a diameter of Ø20 cm, using a liquid molasses-based medium (60 g/L beet molasses, 10 g/L soy peptone). The prepared medium was inoculated with 1% (*v*/*v*) *C. cladosporioides* mould inoculum and poured onto the plates. Cultivation was carried out at 28 °C for 120 h. After cultivation, the moulds were thermally deactivated, filtered, and dried to obtain dry biomass. The biomass yield was determined. The selection criterion for the medium components was based on the highest yield of dry mould mycelium biomass.

### 2.3. Preparation of Biocomposites

The *C. cladosporioides* strain was used to produce fungal biomass, which was reinforced with commercial bleached softwood kraft (BSK) pulp and waste hemp and cotton fibres to produce the biocomposites (Faculty of Material Technologies and Textile Design, Lodz University of Technology). Two types of mycelial biomass were used: mycelium cultivated using the surface method on Petri dishes, and mycelium grown via submerged cultivation in bioreactors. The cotton and hemp fibres used were shredded and cut into small (10 mm ± 2 mm) pieces.

The preparation method included the following steps: (1) cultivation of the mould *Cladosporium cladosporioides*; (2) thermal inactivation of the mycelium at a minimal temperature of 60 °C for 30 min; (3) separation of the mould from the medium; (4) drying of the fungal biomass at temp. 105 °C for 7 h; (5) weighing 30 g of b.d. (bone-dry) softwood kraft pulp, which was then soaked and then defiberised (laboratory pulper, Labor-Meks; Unidrive X 1000, CAT Scientific, Tübingen, Germany). In the first test variant, the BSK pulp was refined separately for 4 min (5800 rpm) at a consistency of 10% in a PFI laboratory beater, according to test method TAPPI/ANSI T 248 sp-21 (PFI mill, Espoo, Finland). After refining, the fungal biomass was added to the pulp. In the second test variant, the dry fungal biomass was weighed, then soaked and added to the BSK pulp before refining. The mixture of BSK pulp and fungal biomass was refined in a PFI laboratory beater for 4 min (5800 rpm) and at a total consistency of 10%. The biocomposite sheets were formed in a Rapid-Köthen machine according to the ISO 5269-2:2004 standard [35] (Rapid-Köthen, Labor-Meks, Lodz, Poland). After forming, the sheets were pressed using a standard 2.8 kg hand roller with a diameter of 140 mm. Pressing was performed under the roller’s own weight in such a way that the roller was rolled three times on the sample surface. Drying was carried out at a temperature of 98 °C and a vacuum pressure of 93 kPa for 6 min. Table 1 presents the composition of the biocomposite materials and the preparation methods.

### 2.4. Evaluation of Material Parameters of Biocomposites

The following material parameters were investigated: apparent density, determined using a Lorentzen & Wettre device, type 222, Sweden (ISO 534:2011 [36]); bursting strength, according to ISO 2758:2014 [37] (Mullen Burst Machine, Lorentzen & Wettre, Kista, Sweden); tearing resistance, determined using an Elmendorf tester (Testing Machines, New Castle, DE, USA, ISO 1974:2012 [38]); SCT compression strength index (SCT index), measured with equipment from Haida International Equipment Co., Ltd., Dongguan, China (ISO 9895:2008 [39]); tensile index and elongation tests, performed using an INSTRON tensile testing machine, model 5564 (High Wycombe, UK, ISO 1924-2:2008 [40]); bending stiffness, measured using the two-point method according to ISO 5628:2019 [41] (TMI Testing Machines Inc., New Castle, DE, USA); air permeance, measured using a Bendtsen tester manufactured by TMI Testing Machines Inc., USA (ISO 5636-3:2013 [42]); and contact angles, measured using a PGX goniometer (TMI Testing Machines, USA) according to the TAPPI T 458 standard method [43].

Because it was difficult to identify the single most important parameter for evaluating the biocomposites, it was assumed that all the examined parameters, except for apparent density, were equally important. Consequently, a multiple-variable optimization method was used to identify the best material. In such cases, it is easiest to present the results through single-criteria optimization, where the objective function returns only one value. For this purpose, the weighted objectives method (WOM) was used. This method involves reducing multi-criteria optimization to a single criterion, by introducing a substitute criterion which is the weighted sum of all criteria, according to the formula

$$\mathrm{WOMR}=\overset{k}{\underset{i=1}{\sum }}w_{i}\cdot x_{i}$$

where

WOMR—weighted objectives method result;

k—number of properties (variables) used in the calculation;

x_i_—normalized value of the property No. “i”;

w_i_—weight value for the property No. “i”.

The values of all weights must be within the range 0–1 and their sum must be equal to 1. Based on these assumptions, eight properties (all except apparent density) were normalized and each of them was given the same weight of 0.125.

### 2.5. Biodegradation of Biocomposites

The study was conducted under two conditions: controlled conditions in pots and field conditions for biocomposites No. 3, 7, 12 and market agrotextile No. 19. The field tests were carried out in class 2 soil (very good arable soil) with a pH of approximately 6.5, in the town of Lubianków (Łódź Voivodeship, coordinates: 51°57′41.746″ N 19°47′49.932″ E). Measurements were taken every 48 h over a period of 14 days. The biocomposites were buried at depths of approximately 10 cm. The percentage of sample degradation was assessed visually.

### 2.6. Application of Biocomposites in Agriculture—Seed Germination

A total of 50 onion and parsley seeds (World of Flowers Sp. z o.o., Warszawa, Poland) were sown in 5 pots, 20 cm in diameter, filled with universal vegetable-growing soil and manure (Ogro-plant, Starcza, Poland). There were 10 seeds of each plant sown in each pot. The seeds were incubated under controlled conditions for 6 weeks. In field conditions, the soil was moistened and then covered with the biocomposites (No. 3, No. 7, and No. 12) or the commercial agrotextile white agricultural fleece (Sumin, Poland) (No. 20). The germination of vegetable seeds (percentage of germinated seeds) was recorded every 24 h from the time of sowing. Seed germination was noted at the moment when the shoots emerged above the soil surface. Sunlight was monitored using a luxometer (Lxp-2 Sonel, Świdnica, Poland) on the following scale: low sunlight “+”—completely overcast, sunlight value below 8000 lux; moderate sunlight “++”—partly cloudy, sunlight value between 8000 and 18,000 lux; high sunlight “+++”—clear sky, sunlight value above 18,000 lux. Soil moisture was measured using a SM150-KIT tester by Geomor Technik, Szczecin, Poland on the following scale: dry soil “+”—topsoil dry and compact below 10% soil moisture; moderate soil moisture “++”—topsoil moist and crumbly, 10–20% soil moisture; high soil moisture “+++”—topsoil wet and sticky, above 20% soil moisture. The soil temperature was measured using a BIOOGRÓD 071905 (Browin, Łódź, Poland) device manufactured in Poland. 

## 3. Results and Discussion

### 3.1. Screening of Moulds

Differences in the amounts of mycelial biomass produced and mycelial growth rates were observed between genera as well as at the strain level (Table 2).

It was noted that strains from the genera *Cladosporium* sp. and *Alternaria* sp. produced significantly more mycelial biomass in submerged culture than the other moulds. The least mycelial biomass was produced by the genera *Aureobasidium* sp., *Fusarium* sp., *Chaetomium* sp., and *Rhizopus* sp. The highest yields of mycelial biomass were found for the strains *Cladosporium* sp. (No. 8) (wet: 33.85 g/dry: 0.71 g), *Ulocladium* sp. (No. 20) (26.20 g/0.58 g), *Alternaria* sp. (No. 2) (24.30 g/0.46 g), and *Cladosporium* sp. (No. 9) (23.20 g/0.60 g). However, the highest growth rates were found for the strains *Penicillium* sp., *Scopulariopsis* sp., and *Cladosporium* sp. (0.0027–0.0034 1/h). Morphological observations of mould growth in submerged cultures showed the formation of compact, coloured pellet structures. In surface cultures, the moulds grew as spore-forming mycelium, with the least abundant growth observed for *Scopulariopsis* sp. (Table 2). Based on the screening study, the strain *Cladosporium cladosporioides* (No. 8), which produces mycelium abundantly and grows rapidly in both submerged and surface cultures, was selected for further study.

The *Cladosporium cladosporioides* strain was found to be the most efficient producer of mycelium. This mould is classified as a saprophyte. It occurs commonly in soils and in all types of ageing and dead plant matter. It is a xerophilic and psychrophilic fungus, as evidenced by its ability to grow in a wide spectrum of humidity and temperature. The optimal growth temperature is 18–28 °C. It is easy to grow in the laboratory. The mycelium can be loose or dense and tends to grow flat [44,45]. It does not show pathogenic features, although the spores can be allergenic and some strains cause grape diseases. It does not produce mycotoxins. These characteristics make mycelium from *Cladosporium cladosporioides* a good candidate for use in the production of biocomposites.

### 3.2. Evaluation of Mould Culture Conditions

The next stage of the study involved the selection of culture conditions for *Cladosporium cladosporioides* (No. 8), taking into account the following parameters: temperature (in the range of 20–28 °C); shaking speed (130–180 rpm); carbon source (sucrose, glucose-fructose syrup, maltodextrins, beet molasses) and concentration (40–100 g/L); nitrogen source (soybean peptone, yeast extract) and concentration (10–50 g/L); and culture type (submerged, surface) under different conditions (Table 3).

The conditions of mould growth have a great influence on the structure and composition of the mycelium cell wall, which in turn can have an impact on the physicochemical properties of the mycelium-bound biocomposite [8,46]. In this study, the biomass yield of *C. cladosporioides* was dependent on the agitation conditions. The highest biomass level in the cultures in Erlenmeyer flasks was obtained with a shaking speed of 180 rpm (6.0 g dry mass/1 L medium). This was confirmed in the submerged culture propagated in a stirred-tank bioreactor at a stirring speed of 350 rpm, where the biomass concentration (equal to 23.7 g/L) was higher than at 200 and 275 rpm (Table 3). A higher mycelial biomass level was reached at 28 °C (4 g/L) than at 20 °C or 25 °C (1.2–2.2 g/L). Of the tested carbon sources, beet molasses provided the highest concentration of mycelial biomass (6.0–11.4 g/L), which was obtained with an initial substrate level of 60 g/l. Much lower biomass production was noted for maltodextrins (2.4–5.8 g/L), glucose-fructose syrup (2.0–4.4 g/L), and sucrose (0.8–3.4 g/L). Yeast extract was found to be a better nitrogen source for *C. cladosporioides* growth than soybean peptone. A yeast extract concentration of 20 g/L resulted in higher biomass levels than 10 g/L and 50 g/L (Table 3). Broth nutrients play an important role in fungal growth because cellular functions are supported by macro- and micronutrients [47]. These nutrients act as energy sources and structural elements, supporting enzyme activity and protein production. It has been proven that high levels of carbon nutrients improve mycelial density, with increased branching and inhibited hyphal extension [11]. The best mycelial growth was achieved with a concentration of 60 g/L beet molasses. The beet molasses comprising various compounds (carbohydrates, lipids, proteins, inorganic compounds) demonstrated higher potential as carbon sources compared to simple nutrient solution components (maltodextrins, glucose-fructose syrup, sucrose). Similarly, Karimi et al. found that moderate amounts of organic and inorganic compounds in industrial by-products enable better mycelial growth than pure chemical compounds at high concentrations [48].

The properties of biocomposites based on mycelial biomass mainly depend on the type of fungus, its growth conditions, the additives used, and the technological processes applied. Similar observations pertain to biocomposites based on waste substrates and mycelia of higher fungi [28,49,50]. The mould *C. cladosporioides* was found to produce mycelium at a relatively high level (23.7 g/L) when cultured in a stirred-tank bioreactor for 120 h at 28 °C and 350 rpm, in a medium composed of beet molasses (60 g/L) and yeast extract (20 g/L) at an initial pH of 4.8 ± 0.2, and with an inoculum ratio of 5% (*v*/*v*) (10^6^ CFU/mL). Half the mycelial yield (11.2 g/L) was obtained in surface culture in the same medium (Table 3). However, given the energy cost in the case of submerged cultures (agitation of the cultivation broth), surface culture can also be considered for obtaining mycelial biomass.

### 3.3. Biocomposites—Technological Aspects and Properties

**Figure 1:**
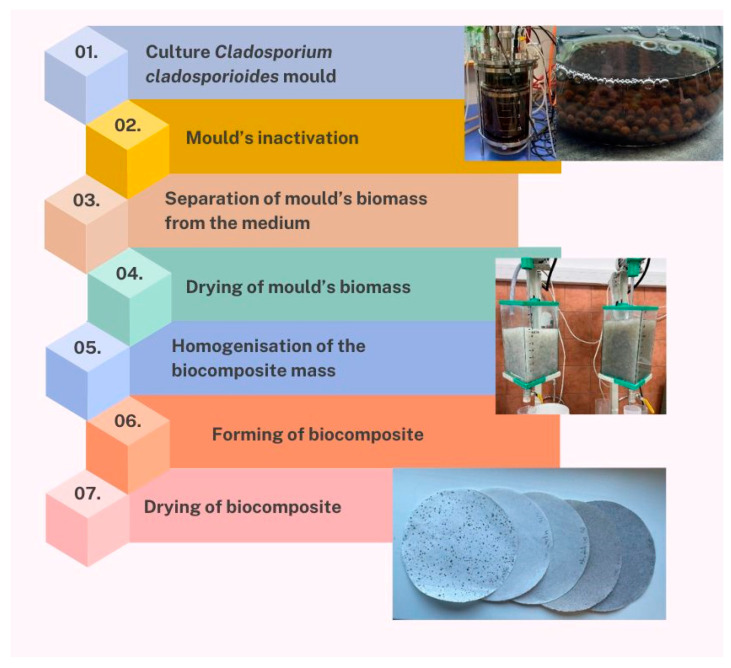
Technological steps in the production of biocomposites based on mycelial biomass and fibrous additives.

A technology was developed for producing biocomposites based on mould biomass with fibre additives (Figure 1).

The first technological step is to culture the mould and obtain mycelium. Then, the mould biomass is inactivated, separated from the medium, and dried. The most important technological step is the homogenization of the biocomposite mass, including dry BSK pulp and dry mould biomass, with the optional addition of hemp/cotton fibres. The mixture of pulps and fibres is refined together and biocomposite sheets are formed under pressure. Finally, the sheets are dried. The technological steps are described in detail in the Section 2.

The proposed technology for producing biocomposites based on mould mycelium and fibre additives leverages the unit operations used in papermaking. The raw material consists of softwood (pine) cellulosic fibres refined with cultured, thermally deactivated mycelium separated from the substrate, as well as non-wood fibre additives (e.g., cotton, hemp). The mixture composed in this way is subjected to dilution and defiberisation. A flat biocomposite is formed and dewatered on a wire. The wet material, still containing approximately 20% dry matter, is pressed and dried under load to obtain a final product of uniform structure. A similar procedure has been applied in previous studies to obtain chitin-nanopaper from higher fungal extracts, using lignocellulosic substrates or wood with fungal glucan extracts [18,19].

The key technological steps in the process are homogenization, forming, pressing, and final drying of the biocomposite. All these steps contribute not only to ensuring a homogeneous material with good mechanical parameters, but also to the release of chitin from the cell wall of the mould mycelium, which improves hydrophobicity. Appels et al. [51] showed that hot pressing, in particular, provides uniform thickness and increases the strength of *T. multicolor* and *P. ostreatus* fungal biocomposites on rapeseed straw substrate, changing their properties from foam-like to cork- and wood-like. The authors demonstrated that by changing the fabrication process, differences in material properties can be obtained. These results confirm that it can be beneficial to use high temperatures in the technological stages of fungal biocomposite production. Esterification, repolymerisation, and hydrogen bond formation at high pressing temperatures are crucial for improving the properties of biocomposites [52]. In another study, heat treatment of chitosan films obtained from *Aspergillus niger* mycelial biomass led to structural reorganisation and reduced solubility [53]. The drying temperature in the final step is also an important factor in the production of biocomposites, as it promotes fungal deactivation and reduces sample contamination [54].

**Figure 2:**
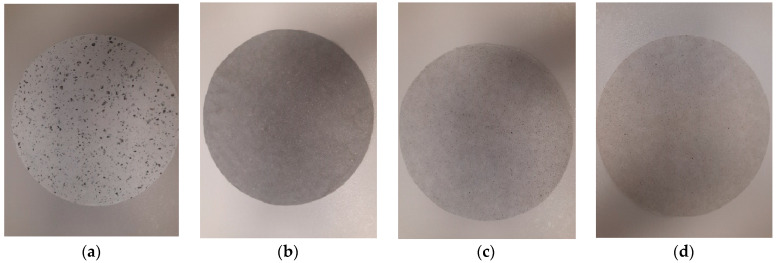
Biocomposites: (**a**) BSK pulp/mycelium (10%), BSK pulp refined separately (No. 1); (**b**) BSK pulp/pellets (40%) refined together (No. 9); (**c**) BSK pulp/mycelium (20%)/cotton fibres (10%) refined together (No. 10); (**d**) BSK pulp/mycelium (20%)/hemp fibres (10%) refined together (No. 13).

To determine the composition of the biocomposite based on mould mycelium and cellulosic fibre additives, 18 variants were produced according to the described method, differing in their ingredients, the way the mycelium was cultured (submerged or surface cultures), and the method of grinding (together or separately) the mycelial biomass and fibre additives. The obtained biocomposites showed different morphologies (Figure 2), especially in the case of biocomposite No. 1, where mycelial biomass was added to the pulp after pulp refining. Small dark spots resulting from mycelial fragments were visible on the surface (Figure 2a). In the case of biocomposites No. 10 and No. 13, where biomasses of pulp, cotton, or hemp fibres and mycelium were refined together, the surface was uniformly light grey, and the surface structure was uniform (Figure 2c,d). When the mycelium was in the form of pellets instead of surface mycelium, the colour of the biocomposite was much darker and the structure was compact (Figure 2b).

The apparent densities of almost all the biocomposites were in the range of 0.54–0.66 g/cm^3^. Only biocomposite No. 11, containing BSK pulp, mycelium (20%), and cotton fibre (20%) had a lower apparent density (0.47 g/cm^3^). This was due to the high amounts of mycelium and cotton fibre additives in this sample (total 40% BSK pulp). In the case of composites containing plant cellulosic fibres, lower density usually means higher porosity, but at the same time also lower strength properties. This relationship is visible in the case of No. 2, in which the BSK pulp and mycelium mass were refined together. The resulting biocomposite was characterized by the highest density (0.66 g/cm^3^) and high strength properties (Table 4).

The bursting strength index was in the range of 4.0–5.8 kPa×m^2^/g for most of the tested samples. The highest bursting strength index was obtained for sample No. 2 (BSK pulp with 10% mycelium refined together) (5.8 kPa×m^2^/g). This value was even higher than the bursting strength index of reference sample No. 14 (containing refined cellulose pulp only: 5.6 kPa×m^2^/g) (Table 4). The lowest bursting strength indexes (3.5–3.9 kPa×m^2^/g) were observed for samples No. 1 (BSK pulp with 10% mycelium masses, BSK pulp refined only), No. 11 (BSK pulp, mycelium 20% and cotton 20%), and No. 16 (BSK pulp with cotton 20%). The addition of 20% cotton resulted in a decrease in bursting strength. The compression resistance (SCT Index) values for samples No. 5 and No. 14 were in the range of 15.9–18.5 N, while the highest values in the range of 24.7–24.8 N were observed for No. 12 (containing 20% mycelium and 10% hemp fibres) and No. 6 (10% mycelium in the form of pellets). The addition of mycelium and waste fibres positively influenced the compression strength properties of the material (Table 4).

Tensile index and elongation are often tested and are extremely important strength properties of fibrous materials. The highest elongation was recorded for biocomposite No. 15, containing cellulose BSK pulp with 10% cotton fibre additive (3.84%). Biocomposite No. 3, containing 20% mould mycelium, showed a similarly high elongation value (3.80%). Increasing the percentage of mycelium improved elongation properties up to a content of 20–30%. Higher mycelium content (40%) resulted in reductions in both elongation and tensile index properties (Table 4). Differences in tearing resistance were observed for the biocomposites made with surface-cultivated mycelium and those made with mycelium cultivated in submerged conditions. Higher values were recorded for biocomposites made with mycelium in pellet form. It was also noted that the addition of waste fibres influenced tearing resistance. The highest tearing resistance was obtained for biocomposites No. 16 (346 mN) and No.11 (332 mN) (Table 4). It can be concluded that the structure of the mycelium pellets combined with BSK pulp improved the material’s integrity. The inclusion of waste fibres also had a positive influence on tear resistance, suggesting these components had a synergistic effect, enhancing the strength of the composite.

Air permeability is an important indicator of the porosity of biocomposite materials (how well air can travel through the material), as well as the degree of bonding between biocomposite components. Very high air permeability values, as seen in biocomposite No. 11 (1529 mL/min), indicate a highly porous material, which allows a large volume of air to pass through, potentially leading to rapid air flow and a lack of thermal insulation. However, the air permeability values for the biocomposites generally ranged from moderate to high (500–1000 mL/min), qualifying them as breathable materials. In general, the addition of mycelium increased air permeability. However, higher permeability was observed for the biocomposites containing both mycelium and waste fibres (600–1500 mL/min) (Table 4). The samples with the highest air permeability were most often characterized by lower apparent density and lower strength properties.

The hydrophobic properties of the produced biocomposites were tested by measuring the contact angles. The static contact angle test demonstrated that the addition of mycelium positively affected the structure of the biocomposite, by limiting the liquid penetration. The highest contact angle was observed in biocomposite No. 10 (86°), which contained 70% BSK pulp, 20% mycelium, and 10% cotton fibres. Finally, in bending resistance tests, the highest stiffness was recorded for biocomposite No. 16 (6.3 mN) (Table 4). The mycelium did not have a clear impact on improving the resistance to deformation.

The addition of 10–30% mycelial biomass (both surface and submerged cultures) and 10–20% hemp or cotton fibre was found to have a positive effect on the properties of the material, especially mechanical properties, static contact angle, and air permeability.

Normalized values for all properties and WOMR values are available in the Supplementary Materials (Table S1). Based on the WOMR analysis, the best material compositions were identified: No. 2, 3, 4, 9, 10, 11, 12, 15, 16. It was observed that the parameters of the produced biocomposite were improved by adding a maximum of 30% mycelium in relation to BSK pulp. It was also found that cotton fibres provided better results than hemp fibres. Similarly, the use of pellets improved the properties of the biocomposite more than the surface-cultured mycelium.

The addition of mycelium was found to improve the hydrophobic properties of the biocomposites, especially when the mycelium was co-milled with other masses. The increased hydrophobicity of the material is related to the presence of chitin in the fungal cell wall. Fungal chitin is significantly more hydrophobic than crustacean chitin [55]. Thus, the addition of fungi to a biocomposite can increase the hydrophobic character of some materials, such as cellulose. These characteristics make fungi-derived materials suitable for a wide range of applications, including coatings, membranes, packaging, and paper. A paper-like biocomposite has been described in the literature, which was obtained from the mycelia of higher fungi (*A. bisporus*, *A. arbuscula*, *M. genevensis*, *T. versicolor*) grown on molasses medium. The authors isolated chitin and glucan polymers from the mycelium and used them to obtain paper. The mechanical and hydrophobic properties (the highest water contact angle was 101–106°) indicated significant improvements over the control paper. The authors attributed these improved properties to the high chitin content [56]. Our biocomposites showed lower hydrophobicity values (max. water contact angle 81–86°), but different conditions were used. The whole mould mycelium was subjected to refining, mixing, pressing, and drying. Chitin and glucan were not extracted, as this is a cost-intensive process.

In the biofabrication of mycelium-associated biocomposites, the choice of substrates also has a significant impact on the process quality, yield, and properties of the biomaterial [1]. In studies by other authors, various additives such as metals, nanoparticles, and plasticisers have been applied to higher fungal mycelium-based biocomposites to obtain useful properties [1]. Appropriate additives (e.g., carboxylated styrene–butadiene rubber latex) can result in a twofold increase in compressive strength compared with dry mycelium [57]. Elsacker et al. found that adding hemp to a higher mycelium-based biocomposite improved the mechanical properties of the material [6]. The authors showed that the size of the hemp fibres is important for improving the compressive modulus of elasticity. Fine hemp fibres (5 mm) improved this parameter about twofold compared with uncut loose fibres. We used small fragments of hemp or cotton waste 10 mm in length. The strength parameters obtained were higher than for the control. Studies by other authors also showed improved properties as a result of the addition of cotton or hemp, and the tested biocomposites based on a higher fungus from the genus *Ganoderma* and fibre additives showed higher bending strengths and flexural moduli compared to the material without additives [26,58].

Selecting the components of substrates to achieve biomaterials with particular properties remains a significant challenge. The use of a single component is simpler and can facilitate biofabrication. On the other hand, a substrate mixture of at least two materials can lead to a more robust product that lasts longer and has better mechanical properties. These properties can be tailored to specific applications, such as lightness for insulation, high density for structural panels, or flexibility in textiles [1].

### 3.4. Biodegradation of Biocomposites

**Figure 3:**
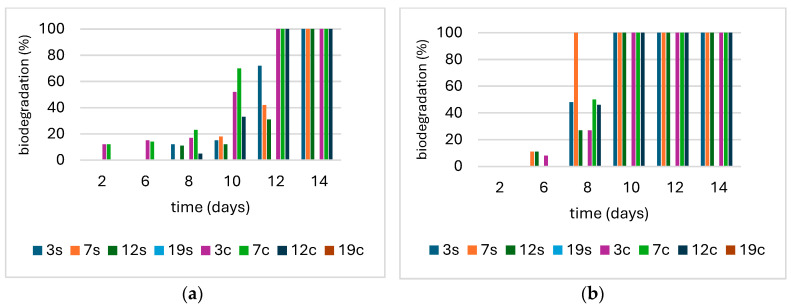
Biodegradation (%) of tested biocomposites (No. 3, 7, 12) and commercial agrotextile (No. 19) in soil (s) and compost (c) under controlled (**a**) and field conditions (**b**).

**Figure 4:**
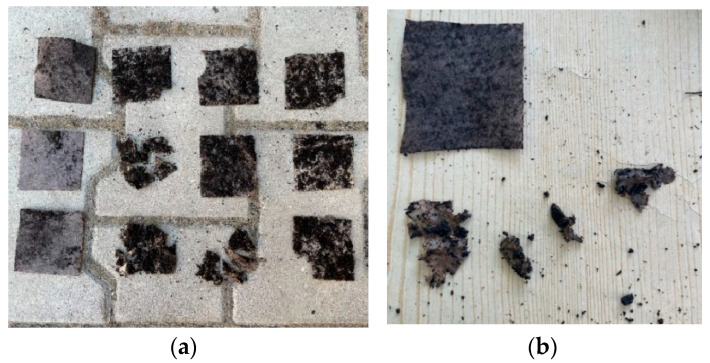
Biodegradation of biocomposite samples. (**a**) Sequentially in rows from top: controlled conditions (soil); controlled conditions (compost); field conditions (soil) at 2 days. In columns from left: agrotextile No. 19, biocomposites No. 3, 7, 12. (**b**) Samples of biocomposites in compost under field conditions after 6 days (only commercial agrotextile sample (No. 19) is not degraded).

The biodegradation of selected biocomposites (No. 3, 7, 12) and, for comparison, commercial agrotextile based on sheep’s wool (No. 19), was investigated in soil and compost under controlled and field conditions. The biocomposites containing mycelial biomass (cultured by submerged and surface methods) and hemp fibres were found to be fully biodegradable. Under controlled conditions in pots, 100% biodegradation was achieved after 14 days in soil; under field conditions, all tested biocomposites were already degraded by day 10. Under controlled conditions, the application of compost accelerated (day 2) and increased the biodegradation level (10–40%) of the obtained biocomposites compared to the soil variant (Figure 3). The commercial agrotextile based on sheep’s wool showed no signs of biodegradation after 20 weeks in any of the experimental variants (Figure 4).

This study confirmed the rapid (10–12 days) biodegradation of the obtained mycelium-based biocomposites in soil, while at the same time, the wool market agrotextile was not biodegradable until week 20. The developed mycelium-based composites consist exclusively of biocompatible components, making them fully biodegradable and not a burden on the environment, similar to mycelium-based bioproducts studied by other authors [59,60]. Previous studies have indicated that after using mycelial biocomposites, they can be composted or re-used, for example as animal supplies, organic fertilisers, soil conditioners, and substrates for seedlings [4,61,62]. An important challenge in the technology of mycelial-based biomaterials is to simultaneously increase the durability of the composites during application while maintaining their biodegradable nature after application, which requires further research [63].

### 3.5. Application of Biocomposites in Agriculture

Under controlled conditions (in pots, stable temperature, humidity, and sunlight), accelerated germination (1–2 weeks) and higher percentages of germinated seeds (2–30%) were observed compared to field conditions (Table 5). The biocomposites also accelerated the germination of onion seeds (1 week) compared to the experiment without seed cover under controlled conditions. Under field conditions, differences were not noticeable. Only the application of biocomposite No. 7 reduced the time of parsley germination by 1 week compared to the control (uncovered seeds). No differences were observed between the results for the biocomposites and the commercial agrotextile. The application of the biocomposites as seed coverings increased the percentage of germinated onion seeds by 10% (biocomposite No. 3, pots, and No. 12, field), 18% (No. 7, field), and 25% (No. 13, field), but had no effect on parsley seeds. Under controlled conditions, the results for onion and parsley seeds were comparable, while under field conditions the wool fibre agrotextile increased the percentage of germinated seeds by 30–40% compared to the developed biocomposites. Overall, biocomposite No. 3 showed the best results, accelerating onion seed germination by 1 week and increasing the percentage of germinated seeds by 10% under control conditions and 25% under field conditions (Table 5).

The seed germination studies confirmed the possibility of using the produced biomaterial as agrotextiles in horticulture. Mycelium biocomposites have not been used previously for this purpose. For the mycelial biomass composites to achieve wide applicability and effectively replace existing market solutions, several key factors must be addressed. These include reducing production costs, scaling up from laboratory to industrial production with efficient automation, and establishing certification standards to ensure product safety and reliability [63]. Rapid technological advancements in mycelium-based materials and growing market demand for sustainable products position mycelium composites as a highly promising biomaterial with the potential for diverse new applications.

The investigated biocomposites, based on waste mould mycelium and fibre materials, have potential applications in the textile industry as agrotextiles and in the pulp and paper industry as modified paper. To date, the most extensively studied biocomposites have a higher content of fungal mycelium and are predominantly applied in the textile and leather industries. Their uses include clothing, footwear, and bags, owing to their unique manufacturing process (overgrowth of lignocellulosic waste materials) as well as their favourable structural properties (hardness and consistency) and mechanical properties. [15]. The biocomposite solution presented in this study, based on mould biomass and waste fibres, is characterized by a more homogeneous, finer structure, similar to that of a textile or paper.

## 4. Conclusions

Many biotechnology industries rely on moulds for the production of antibiotics, foods, acids, enzymes, and other products. This results in significant waste, in the form of unused mycelial biomass. Innovations in materials technology based on higher fungi have enabled the development of new biocomposites with interesting properties. However, the potential for creating biocomposites using mould biomass has not been explored previously. In this study, we developed a novel biocomposite based on mould biomass and waste fibres, beginning with the selection of mould species and optimization of culture conditions. The biocomposite was produced using a paper-making method, followed by comprehensive characterization and evaluation of its application as an agrotextile fabric. Throughout the experiments, the focus was on adhering to sustainability principles, utilizing waste-based solutions, and creating a biodegradable material with functional properties. The results indicate that mould mycelium with wood pulp and fibre additives (cotton, hemp) can serve as a base for biocomposites that offer a sustainable alternative to synthetic agrotextiles. Due to its chemical composition (chitin, glucans), mycelium increases the hydrophobicity and biodegradability of the material. Additionally, technological processes using high temperature, homogenization with other fibrous additives, grinding, and pressing promote the release of chitin, ensuring structural homogeneity and enhancing its mechanical properties. Future work should focus on refining the production process and tailoring the composition of biocomposites based on mould biomass and waste fibres for diverse industrial applications.

## Figures and Tables

**Table 1:** Biocomposite variants produced in the studies.

No.	Composition		Preparation of the Biocomposites
	Mould Biomass	Fibre Materials	
1	mycelium 3 g (10% dry mass)	BSK pulp 30 g (b.d.)	mould culture on the medium surface; pulp refined only data; biomass added to pulp after pulp refining
2	mycelium 3 g (10%)	BSK pulp 30 g (b.d.)	mould culture on the medium surface; biomass and fibres refined together
3 #*	mycelium 6 g (20%)	BSK pulp 30 g (b.d.)	mould culture on the medium surface; biomass and fibres refined together
4	mycelium 9 g (30%)	BSK pulp 30 g (b.d.)	mould culture on the medium surface; biomass and fibres refined together
5	mycelium 12 g (40%)	BSK pulp 30 g (b.d.)	mould culture on the medium surface; biomass and fibres refined together
6	pellets 3 g (10%)	BSK pulp 30 g (b.d.)	mould culture in a bioreactor; biomass and fibres refined together
7 #*	pellets 6 g (20%)	BSK pulp 30 g (b.d.)	mould culture in a bioreactor; grinding of biomass and fibres together biomass and fibres refined together
8	pellets 9 g (30%)	BSK pulp 30 g (b.d.)	mould culture in a bioreactor; biomass and fibres refined together
9	pellets 12 g (40%)	BSK pulp 30 g (b.d.)	mould culture in a bioreactor; biomass and fibres refined together
10	mycelium 6 g (20%)	BSK pulp 30 g (b.d.) dry mass cotton fibre 3 g (10%)	mould culture on the medium surface; biomass and fibres refined together
11	mycelium 6 g (20%)	BSK pulp 30 g (b.d.) dry mass cotton fibre 6 g (20%)	mould culture on the medium surface; biomass and fibres refined together
12 #*	mycelium 6 g (20%)	BSK pulp 30 g (b.d.) dry mass hemp fibre 3 g (10%)	mould culture on the medium surface; biomass and fibres refined together
13	mycelium 6 g (20%)	BSK pulp 30 g (b.d.) dry mass hemp fibre 6 g (20%)	mould culture on the medium surface; biomass and fibres refined together
14	-	BSK pulp 30 g (b.d.)	control sample
15	-	BSK pulp 30 g (b.d.) dry mass cotton fibre 3 g (10%)	control sample
16	-	BSK pulp 30 g (b.d.) dry mass cotton fibre 6 g (20%)	control sample
17	-	BSK pulp 30 g (b.d.) dry mass hemp fibre 3 g (10%)	control sample
18	-	BSK pulp 30 g (b.d.) dry mass hemp fibre 6 g (20%)	control sample
19 #	agrotextile; natural fibres; sheep’s wool	control sample (purchased at the market, Agrimpex, Jarosław, Poland)	
20 *	agrotextile; synthetic fibres; polypropylene	control sample (purchased at the market, Sumin, Wargowo, Poland)	

* Biocomposite and agrotextile samples used in agricultural research (seed germination: agrotextile Sumin, Poland, polypropylene fibres) and # biodegradation tests in soil (agrotextile Agrimpex, Poland, natural sheep’s wool fibres); “-” indicates samples without mycelium biomass; b.d.—bone dry.

**Table 2:** Screening of mould strains.

No.	Moulds	Mycelium Biomass Wet/Dry [g]	Growth Rate μ Max [1/h]	Photos of Mould Growth in Submerged/Surface Cultures
1	*Alternaria* sp.	20.85/0.33	0.0019	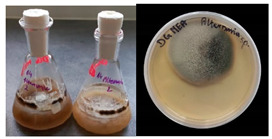
2	*Alternaria* sp.	24.30/0.46	0.0014	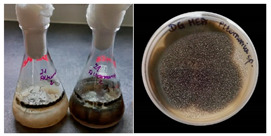
3	*Aspergillius flavus*	14.29/0.42	0.0013	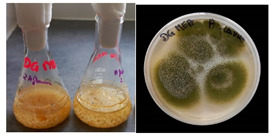
4	*Aspergillius fumigatus*	18.44/0.45	0.0014	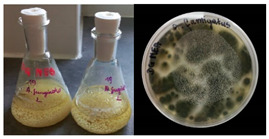
5	*Aspergillius niger*	8.33/0.24	0.0016	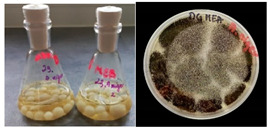
6	*Aureobasidium* sp.	6.04/0.31	0.0007	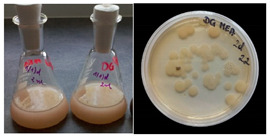
7	*Aureobasidium* sp.	5.48/0.28	0.0010	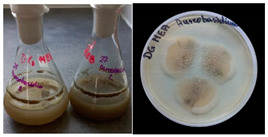
8 *	*Cladosporium* sp.	33.85/0.71	0.0015	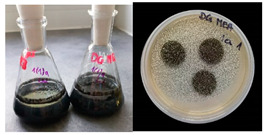
9	*Cladosporium* sp.	23.20/0.60	0.0027	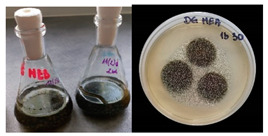
10	*Chaetomium globosum*	11.96/0.24	0.0009	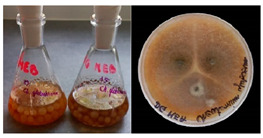
11	*Chaetomium* sp.	8.38/0.22	0.0012	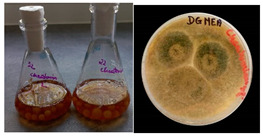
12	*Fusarium* sp.	9.98/0.45	0.0012	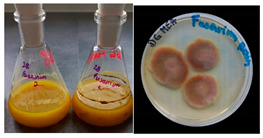
13	*Fusarium oxysporum*	6.02/0.38	0.0008	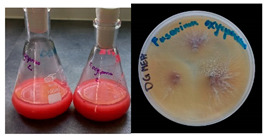
14	*Mucor* sp.	13.23/0.43	0.0018	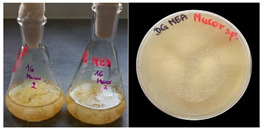
15	*Penicillium* sp.	13.83/0.47	0.0034	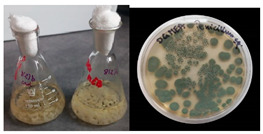
16	*Penicillium* sp.	19.07/0.52	0.0031	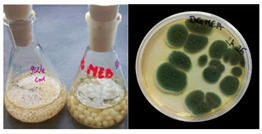
17	*Rhizopus* sp.	9.44/0.35	0.0008	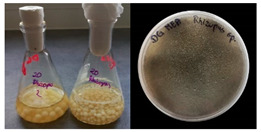
18	*Scopulariopsis* sp.	11.28/0.40	0.0030	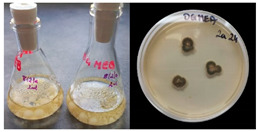
19	*Trochoderma viride*	12.61/0.45	0.0014	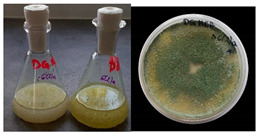
20	*Ulocladium* sp.	26.20/0.58	0.0026	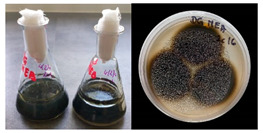

* Strain selected after screening.

**Table 3:** Selection of culture conditions for mould *Cladosporium cladosporioides*.

Culture Conditions	Culture Parameter Tested	Culture Variants		Dry Mass of Mycelium per Sample [g]	Dry Mass of Mycelium per Medium [g/L]
Medium: MEB Culture type: submerged Shaking speed: 190 rpm	Temperature [°C]	20		0.06 ± 0.01	1.2
		25		0.11 ± 0.02	2.2
		28		0.20 ± 0.02	4.0
Medium: MEB Temp. 28 °C Culture type: submerged	Shaking speed [rpm]	130		0.11 ± 0.02	2.2
		160		0.20 ± 0.00	4.0
		180		0.30 ± 0.02	6.0
Nitrogen source in the medium: casein peptone tryptone 5 g/L Temp. 28 °C Culture type: submerged Shaking speed: 190 rpm	Carbon source in the medium [g/L]	Sucrose	40	0.04 ± 0.03	0.8
			60	0.10 ± 0.04	2.0
			100	0.17 ± 0.01	3.4
		Glucose-fructose syrup	40	0.10 ± 0.03	2.0
			60	0.15 ± 0.01	3.0
			100	0.22 ± 0.03	4.4
		Maltodextrins	40	0.12 ± 0.02	2.4
			60	0.17 ± 0.01	3.4
			100	0.29 ± 0.03	5.8
		Beet molasses	40	0.30 ± 0.28	6.0
			60	0.57 ± 0.07	11.4
			100	0.44 ± 0.06	8.8
Carbon source in the medium: beet molasses 60 g/L Temp. 28 °C Culture type: submerged Shaking speed: 190 rpm	Nitrogen source in the medium [g/L]	Soybean peptone	10	0.12 ± 0.01	2.4
			20	0.14 ± 0.02	2.8
			50	0.18 ± 0.04	3.6
		Yeast extract	10	0.10 ± 0.01	2.0
			20	0.21 ± 0.01	4.2
			50	0.20 ± 0.00	4.0
Carbon source in the medium: beet molasses 60 g/L Nitrogen source in the medium: soy peptone 10 g/L Temp. 28 °C	Culture type	Bioreactor culture 1	200 rpm	1.60 ± 0.10	6.7
		Bioreactor culture 2	275 rpm	2.05 ± 0.50	9.2
		Bioreactor culture 3	350 rpm	3.10 ± 3.60	23.7
		Surface culture	Without agitation	2.23 ± 0.11	11.2

**Table 4:** Properties of biocomposites. Coefficient of variation (COV) values are given in brackets (%).

No	Apparent Density [g/cm^3^]	Bursting Strength Index [kPa×m^2^/g]	Index SCT [N]	Tensile Index [N·m/g]	Elongation [%]	Tearing Resistance [mN]	Air Permeability [mL/min]	Static Contact Angle [°]	Bending Resistance [mN]
1	0.53 (4.7)	3.9 (17.0)	15.9 (13.0)	58.0 (8.8)	2.50 (18.8)	147 (1.3)	714 (12.3)	45.9 (28.5)	3.3 (0)
2	0.66 (4.3)	5.8 (5.2)	22.0 (8.5)	79.3 (6.6)	3.40 (10.5)	187 (1.3)	513 (11.4)	77.0 (9.8)	3.0 (0)
3	0.64 (1.7)	5.1 (7.2)	21.2 (13.7)	70.3 (5.1)	3.80 (6.0)	219 (4.8)	674 (9.1)	72.7 (5.3)	4.5 (12.8)
4	0.65 (1.6)	5.2 (9.2)	20.4 (18.1)	70.8 (11.2)	3.75 (12.0)	170 (5.3)	632 (14.2)	77.8 (9.6)	3.3 (15.4)
5	0.59 (2.3)	4.4 (8.1)	18.5 (9.4)	54.8 (7.3)	3.31 (9.8)	173 (13.3)	914 (13.7)	44.9 (12.3)	3.5 (16.5)
6	0.65 (1.3)	4.7 (13.5)	22.2 (10.4)	66.1 (3.9)	3.32 (7.5)	232 (4.1)	505 (14.2)	69.8 (13.5)	4.0 (35.3)
7	0.64 (2.1)	4.7 (12.7)	20.9 (13.0)	69.2 (4.8)	3.32 (9.6)	203 (13.8)	758 (16.6)	54.9 (21.1)	3.0 (0)
8	0.62 (2.3)	4.9 (9.1)	20.9 (7.7)	72.9 (3.5)	3.60 (7.5)	244 (11.0)	751 (12.7)	62.8 (30.8)	3.0 (0)
9	0.63 (2.4)	4.7 (6.2)	21.2 (9.3)	59.3 (7.8)	3.2 (7.5)	234 (3.4)	800 (13.8)	81.0 (6.5)	5.3 (39.3)
10	0.58 (4.6)	4.0 (9.1)	21.1 (8.4)	53.5 (7.9)	3.31 (30.7)	261 (8.4)	1335 (13.2)	86.0 (3.19)	3.0 (0)
11	0.47 (3.9)	3.5 (10,5)	20.4 (8.8)	46.1 (11.8)	2.84 (10.1)	332 (16.2)	1529 (11.3)	74.2 (11.1)	3.3 (13.3)
12	0.60 (1.9)	4.6 (8.7)	24.8 (8.2)	58.7 (7.7)	3.22 (8.0)	267 (27.5)	701 (7.9)	81.1 (6.9)	5.7 (28.5)
13	0.59 (1.3)	4.2 (8.7)	22.6 (13.2)	54.6 (12.6)	2.92 (14.3)	303 (6.7)	628 (8.8)	56.6 (12.9)	3.0 (0.0)
14	0.64 (2.2)	5.6 (4.6)	18.0 (12,3)	73.5 (4.0)	3.18 (8.1)	149 (15.5)	482 (17.8)	42.1 (14.3)	3.3 (15.4)
15	0.60 (3.1)	4.2 (10.8)	22.3 (8.2)	59.3 (6.1)	3.84 (44.9)	260 (45,6)	956 (9.4)	63.6 (11.9)	4.3 (38.5)
16	0.55 (4.6)	3.6 (10.2)	20.8 (11.9)	52.7 (8.1)	2.59 (10.7)	346 (6.3)	1353 (12.4)	55.7 (10.0)	6.3 (21.6)
17	0.60 (2.1)	4.1 (6.6)	21.7 (9.4)	52.7 (7.7)	2.94 (10.8)	260 (21.4)	748 (13.6)	36.2 (12.5)	5.0 (40.0)
18	0.61 (2.3)	4.0 (9.8)	23.8 (6.7)	55.9 (8.5)	2.85 (7.0)	344 (11.8)	472 (6.5)	48.2 (8.8)	4.5 (33.3)

**Table 5:** Germination of seeds under controlled and field conditions using tested biocomposites and market agrotextile.

Variants: Vegetable/ Biocomposite	Controlled Conditions							Field Conditions						
	Conditions	% of Germinated Seeds in the Following Weeks						Conditions	% of Germinated Seeds in the Following Weeks					
		1	2	3	4	5	6		1	2	3	4	5	6
Control, Onion/without biocomposite	T: 7–17 °C; M: (+/++); S: (+)	0 ± 0	0 ± 0	9 ± 7	44 ± 7	75 ± 1	76 ± 0	T: 5–21 °C; M: (++); S: (++/+++)	0 ± 0	0 ± 0	6 ± 3	14 ± 5	31 ± 1	34 ± 0
Control, Parsley/without biocomposite	T: 7–17 °C; M: (+/++); S: (+)	0 ± 0	0 ± 0	7 ± 4	41 ± 16	77 ± 8	84 ± 1	T: 5–21 °C; M: (++); S: (++/+++)	0 ± 0	0 ± 0	0 ± 0	17 ± 12	28 ± 0	28 ± 0
Onion/biocomposite 3	T: 7–17 °C; M: (+++); S: (+)	0 ± 0	5 ± 3	34 ± 14	66 ± 5	83 ± 4	86 ± 0	T: 6–21 °C; M: (++); S: (++/+++)	0 ± 0	0 ± 0	2 ± 0	29 ± 7	52 ± 3	59 ± 1
Parsley/biocomposite 3	T: 7–17 °C; M: (+++); S: (+)	0 ± 0	0 ± 0	2 ± 1	22 ± 19	63 ± 9	72 ± 0	T: 6–21 °C; M: (++); S: (++/+++)	0 ± 0	0 ± 0	0 ± 0	16 ± 7	22 ± 3	24 ± 0
Onion/biocomposite 7	T: 8–17 °C; M: (+++); S: (+)	0 ± 0	5 ± 1	25 ± 10	53 ± 7	69 ± 7	76 ± 0	T: 6–21 °C; M: (++); S: (++/+++)	0 ± 0	0 ± 0	6 ± 5	32 ± 1	50 ± 3	52 ± 0
Parsley/biocomposite 7	T: 8–17 °C; M: (+++); S: (+)	0 ± 0	0 ± 0	0 ± 0	15 ± 11	58 ± 15	72 ± 0	T: 6–21 °C; M: (++); S: (++/+++)	0 ± 0	0 ± 0	3 ± 1	9 ± 1	13 ± 3	18 ± 0
Onion/biocomposite 12	T: 8–16 °C; M: (+++); S: (+)	0 ± 0	9 ± 3	31 ± 11	47 ± 1	54 ± 4	58 ± 0	T: 6–19 °C; M: (++); S: (++/+++)	0 ± 0	0 ± 0	5 ± 3	23 ± 1	37 ± 9	42 ± 0
Parsley/biocomposite 12	T: 8–16 °C; M: (+++); S: (+)	0 ± 0	0 ± 0	7 ± 1	25 ± 10	64 ± 4	70 ± 0	T: 6–19 °C; M: (++); S: (++/+++)	0 ± 0	0 ± 0	0 ± 0	5 ± 3	16 ± 3	19 ± 1
Onion/market agrotextile 20	T: 7–17 °C; M: (+++); S: (+)	0 ± 0	4 ± 2	19 ± 7	38 ± 6	54 ± 2	56 ± 0	T: 6–19 °C; M: (++); S: (++/+++)	0 ± 0	0 ± 0	6 ± 5	36 ± 7	70 ± 9	78 ± 0
Parsley/market agrotextile 20	T: 7–17 °C; M: (+++); S: (+)	0 ± 0	0 ± 0	4 ± 3	24 ± 16	62 ± 9	72 ± 0	T: 6–19 °C; M: (++); S: (++/+++)	0 ± 0	0 ± 0	0 ± 0	23 ± 15	53 ± 4	60 ± 0

T—air temperature [°C]; M—soil moisture: “+” low, <10%, “++”, medium, 10–20%, “+++” high, >20%; S—insolation: “+” low, completely overcast, insolation value ≤8000 lux, “++”, medium, overcast with glare, insolation value 8000–18,000 lux, “+++” high, no cloudiness, insolation value ≥ 18,000 lux.

## Data Availability

The original contributions presented in this study are included in the article and Supplementary Materials. Further inquiries can be directed to the corresponding author.

## Supplementary Materials

The following supporting information can be downloaded at https://www.mdpi.com/article/10.3390/ma17246084/s1, Table S1: Normalized values of all properties included in the WOM analysis and the results (WOMR). The highest values for each property and the seven best materials are marked in green. The product and technology are protected by a patent application submitted to the Polish Patent Office titled “Biodegradable agrotextile based on mould mycelium with fibre mass additives, and the method of its production”, No. P.450149 (28 October 2024).
