# Supplementary material for: Biocomposites Based on Mould Biomass and Waste Fibres for the Production of Agrotextiles: Technology Development, Material Characterization, and Agricultural Application
Source: Materials (Basel). 2024 Dec 12;17(24):6084. doi: 10.3390/ma17246084 (PMC11728375; doi:10.3390/ma17246084)
Supplement: Supplementary file 1 [file materials-17-06084-s001.zip › materials-3325890-supplementary.pdf]

Table S1. Normalized values of all properties included in the WOM analysis and the results (WOMR). The highest values for each property and the seven best materials are marked in green.

No	Bursting strength index [kPa×m²/g]	Index SCT [N]	Tensile index [N·m/g]	Elongation [%]	Tearing resistance [mN]	Air permeability [mL/min]	Static contact angle [°]	Bending resistance [mN]	WOMR
1	0.174	0	0.358	0	0	0.229	0.195	0.091	0.131
2	1	0.685	1	0.672	0.201	0.039	0.819	0.000	0.552
3	0.696	0.596	0.729	0.970	0.362	0.191	0.733	0.455	0.591
4	0.739	0.506	0.744	0.933	0.116	0.151	0.835	0.091	0.514
5	0.391	0.292	0.262	0.604	0.131	0.418	0.175	0.152	0.303
6	0.522	0.708	0.602	0.612	0.427	0.031	0.675	0.303	0.485
7	0.522	0.562	0.696	0.612	0.281	0.271	0.376	0.000	0.415
8	0.609	0.562	0.807	0.821	0.487	0.264	0.534	0.000	0.511
9	0.522	0.596	0.398	0.522	0.437	0.310	0.900	0.697	0.548
10	0.217	0.584	0.223	0.604	0.573	0.816	1	0	0.502
11	0	0.506	0	0.254	0.930	1	0.763	0.091	0.443
12	0.478	1	0.380	0.537	0.603	0.217	0.902	0.818	0.617
13	0.304	0.753	0.256	0.313	0.784	0.148	0.410	0.000	0.371
14	0.913	0.236	0.825	0.507	0.010	0.009	0.118	0.091	0.339
15	0.304	0.719	0.398	1	0.568	0.458	0.550	0.394	0.549
16	0.043	0.551	0.199	0.067	1	0.833	0.392	1	0.511
17	0.261	0.652	0.199	0.328	0.568	0.261	0	0.606	0.359
18	0.217	0.888	0.295	0.261	0.990	0	0.241	0.455	0.418
